# The Alignment of the Tibial Component in Total Knee Arthroplasty: Is a Technology-Assisted System Better Than Conventional Instrumentation?

**DOI:** 10.7759/cureus.54745

**Published:** 2024-02-23

**Authors:** Daniel Hernandez-Vaquero, Alfonso Noriega-Fernández, Sergio Roncero-González

**Affiliations:** 1 Department of Surgery, School of Medicine, University of Oviedo, Oviedo, ESP; 2 Department of Orthopedics and Traumatology, Hospital Universitario San Agustin, Avilés, ESP

**Keywords:** extramedullary alignment, intramedullary alignment, frontal tibial axis, tibial component, computer-assisted, technologies, angulation, total knee arthroplasty

## Abstract

Background

Technologies such as navigation and robotics are aimed at improving tibial alignment in total knee arthroplasties (TKA) and eliminating the errors resulting from the use of manual instrumentation.

Methods

This prospective study analyzed 130 arthroplasties in order to determine whether navigation can improve the frontal mechanical axis of the tibia and whether the postoperative angulation of this axis differs from the preoperative one. The mean patient age was 71.8 years, and the mean BMI was 31.17. Eighty-six patients were female. The same cemented TKA model and the same imageless navigation system were used in all cases.

Results

The mean postoperative tibial angle following implantation was 87.65°, without any statistically significant differences with respect to the previous angulation. However, navigation was seen to result in a nearly neutral tibial axis, a larger number of cases (41.5%-60.8% {p = 0.002}) aligned within the safe zone (90° ± 3°), a smaller number of outliers, and a clustering of values around the mean.

Conclusions

Navigation improves the frontal positioning of the tibial component in total knee arthroplasties but does not offer any advantages as compared with conventional instrumentation.

## Introduction

Total knee arthroplasty (TKA) is considered one of the surgical procedures offering the best medium- and long-term clinical results, with survival rates above 80% at 25 years [1]. One of the alleged prerequisites for these good results is the correct placement of the prosthetic components, following the axis of the limb. As far as the tibial component is concerned, it should be aligned following the mechanical axis of the tibia and placed in the so-called safe zone, leaving an angle of 90° ± 3° between the mechanical axis and the surface of the proximal tibia [2]. Surgeons can nowadays avail themselves of several technological aids to achieve the correct placement of the prosthesis, ranging from manual instrumentation to navigation/robotics systems. Manual instrumentation may be intra- or extramedullary, depending on whether the alignment of the tibial axis is determined based on the position of the medullary canal or a series of external anatomical landmarks.

Computer-assisted technologies provide the surgeon with information on the range of motion, size, stability, and ligament balance of the knee, as well as on the presence of potential deformities. They also ensure the constant and continuous monitoring of all surgical maneuvers. Moreover, they provide a preoperative picture of the mechanical axis of the limb without the need for previous imaging studies. Following the implantation of the TKA, surgeons can ascertain the correct orientation of the limb axis. To ensure the correct placement of the tibial component, navigation systems use the palpation of certain anatomical areas combined with the mapping of the tibial surface. As no intramedullary maneuvers are required, the technique can be compared with the use of extramedullary manual instrumentation, although in navigated procedures the alignment of the axis is obtained from a previously determined algorithm while in the extramedullary technique it depends exclusively on the surgeon’s judgement. This being said, the information sent to the computer is based on the collection of purely subjective landmarks, whose reliability is often questionable given that their uniformity, consistency, and invariability have not been determined.

Historically, two manual techniques have been used to determine the tibial axis and facilitate the placement of the tibial component in TKA: the intramedullary and the extramedullary techniques. Despite the large amounts of studies published in the literature, there are not enough grounds to favor one technique over the other. The intramedullary technique has its opponents, with complications such as fat embolisms and iatrogenic fractures having been described. The authors have also warned of the difficulties inherent in using such techniques in the context of narrow medullary canals or severe tibial deformities caused by bone conditions, old fractures, or the presence of fixation hardware. With the extramedullary technique, the guide must be placed on the anterior tuberosity, at the patellar ligament, and at the center of the ankle, its greatest weakness stemming from inconsistencies in the identification of landmarks, their variability, and their subjectivity.

The main goal of this study was to investigate whether navigation can change the original axis of the tibia and whether such a change is related to the patient’s previous deformity. Also, we set about determining whether navigation is more effective than conventional extramedullary instrumentation in guiding the placement of the prosthetic tibial component. Our goals were therefore as follows: (1) determining whether navigation improves the alignment of the frontal mechanical axis of the tibia following the implantation of a TKA; (2) evaluating whether any differences existed, taking into consideration the patient’s previous deformity; and (3) comparing the tibial alignment achieved with navigation surgery with that reported in the literature obtained using manual extramedullary instrumentation.

## Materials and methods

**Figure 1:**
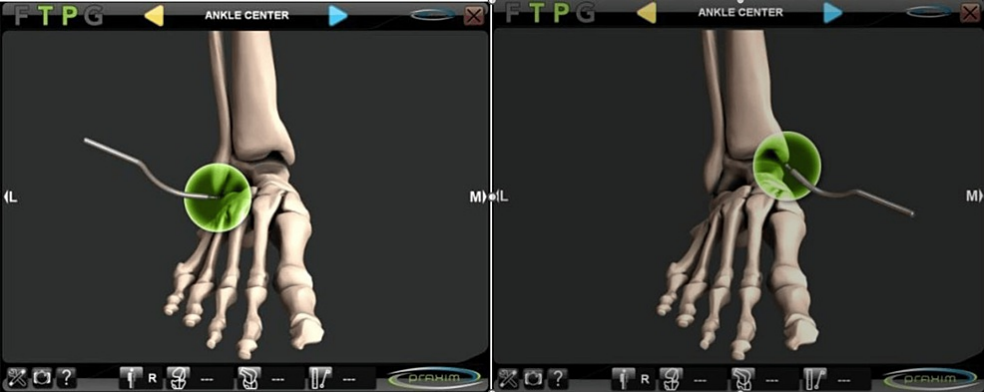
Use of a pointer to identify landmarks in the medial and lateral malleoli of the ankle

This was a prospective study of 130 cases of navigated TKA. The mean patient age was 71.8 years (SD: 9.53), and the mean BMI was 31.17 (SD: 5.55). Eighty-six patients were female. The same cemented TKA model (Apex TKR, Corin Group, Gloucestershire, the United Kingdom) and the same imageless navigation system (Nanostation, Total Knee Surgetics, Praxim, SA, La Tronche, France) were used in all cases. During navigation, tibial alignment was determined, first of all, by placing a bicortical pin 3 cm below the proximal tibial surface. The center of the ankle was determined by palpation, placing the pointer on the most prominent point of the fibular and tibial malleoli (Figure 1).

**Figure 2:**
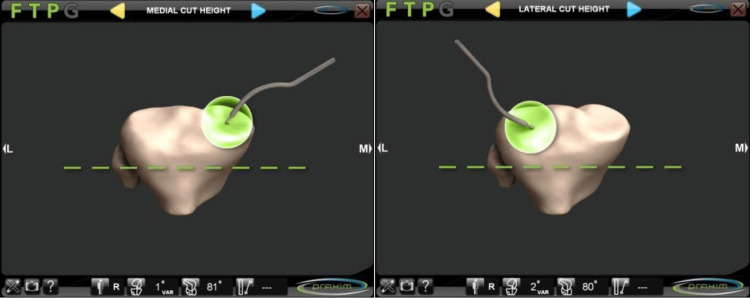
Referencing of the medial and lateral borders of the tibial tray Depth of the tibial plateau

**Figure 3:**
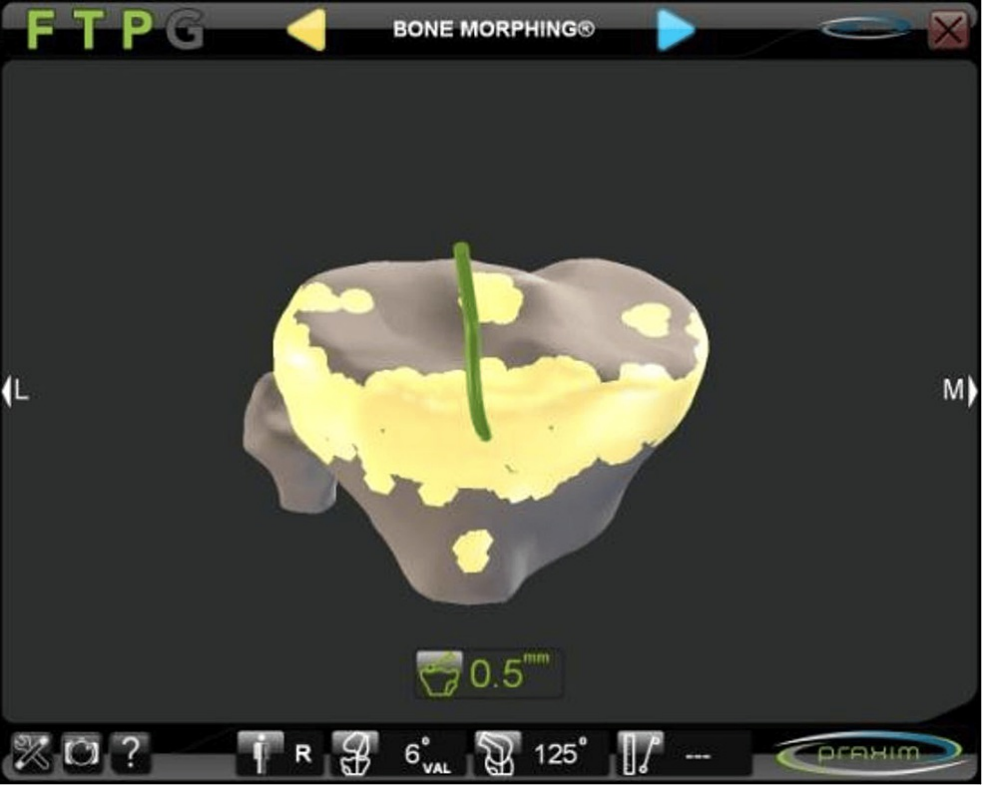
Mapping of the tibial surface

The center of the proximal tibial surface was established by palpating the tibial spine and the tibial tuberosity and by mapping the surfaces of the medial and lateral tibial plateau (Figures 2, 3).

**Figure 4:**
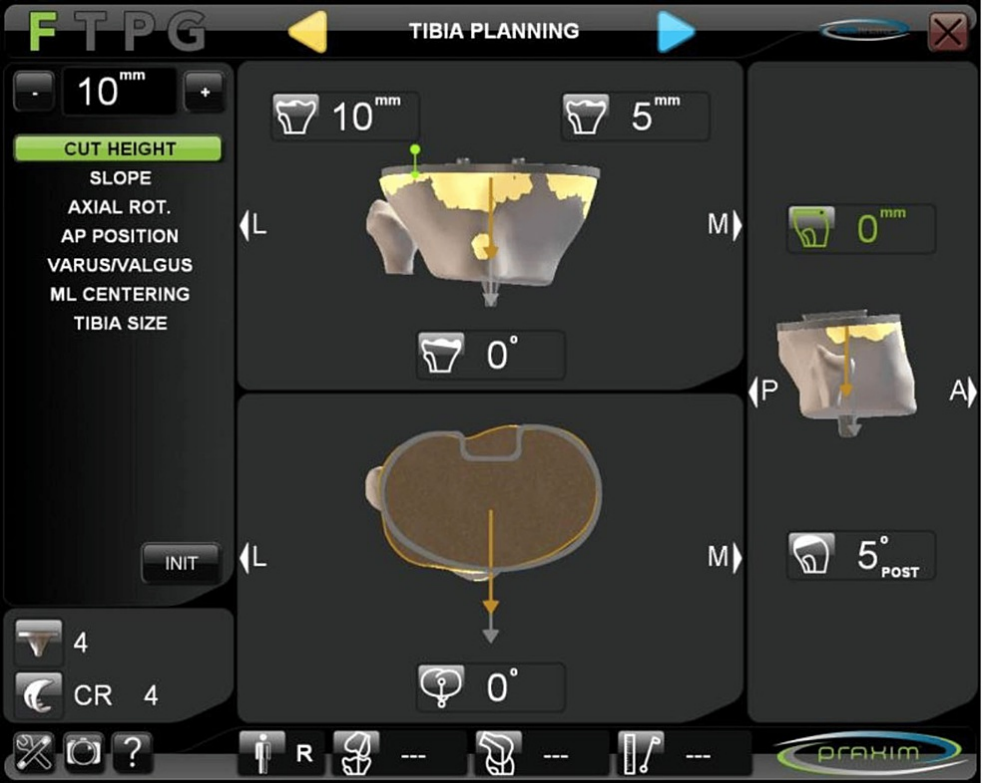
Templating of the position and size of the tibial component AP, anteroposterior; ML, mediolateral

Once this data was entered into the system, an algorithm was used to construct a virtual image that guided the surgeon as to the most appropriate size and spatial position of the implant (Figure 4).

Long X-rays including the hip, the knee, and the ankle were performed before and immediately after the procedure, with the knee fully extended and the patella at the center of the femoral groove. A marker of known size was placed in the vicinity of the knee to ensure the accurate measurement of all angles and distances and of the size of bone structures. The images were stored in the system’s picture archiving and communication system (PACS) and sent to a preoperative planning software (Agfa Orthopaedics Tools version 2.06, Agfa HealthCare, Mortsel, Belgium) using the IMPAX version 6.3.1.2813 visualization package (Agfa HealthCare, Mortsel, Belgium). By drawing a line from the center of the ankle to the center of the upper tibial surface and another line running parallel to the proximal tibial surface, it was possible to determine the preoperative frontal angle of the tibia, in accordance with the landmarks published in the literature [3,4]. The measurements were made by two of the authors, who were in the habit of using the abovementioned preoperative planning software. The postoperative tibial angle was determined by measuring the tibial component angle (TCA) drawing a line from the center of the knee to the ankle and another line running above the prosthetic tibial tray. The TCA was varus if larger than 90º and valgus if smaller than 90°.

All patients were provided with detailed information about the characteristics of the study and were asked to provide their informed consent prior to their enrolment. The study was approved by the Regional Ethics Committee of Asturias, Spain (approval code: PI12/01098).

## Results

An analysis of the preoperative X-rays of the affected limb revealed that 83 patients presented with a deformity, 37 of them with valgus deformities (p < 0.001). Females presented with more deformities than males (p < 0.465), and varus deformities of the mechanical tibiofemoral axis were more common among patients with a high BMI.

**Figure 5:**
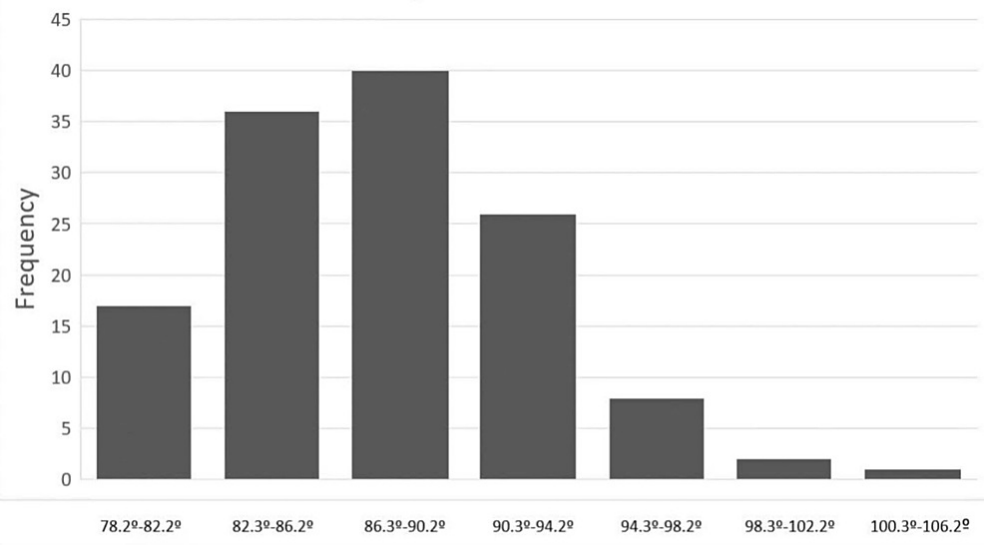
Tibial axis before surgical intervention Tendency toward a valgus deformity

**Figure 6:**
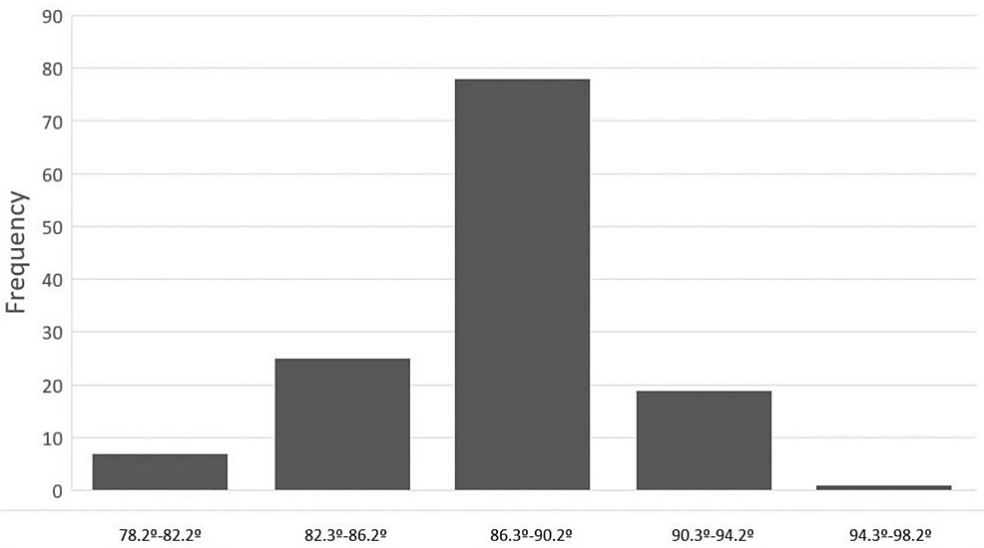
Tibial axis after knee arthroplasty with navigation The tibial angle approaches normality

As far as the tibia was concerned, it was preoperatively determined that the largest and the smallest tibial angles were 102.8° and 78.21°, respectively, with a mean value of 87.66° (SD: 4.7°). The largest and the smallest TCAs were 95.2° and 78.2°, respectively, on the preoperative radiographs, with a mean of 87.65° (SD: 2.79°). No statistically significant differences were found between pre- and postoperative measurements (p = 0.99), which means that navigation did not change the mean preoperative tibial axis. It was however observed that the tibial axis did show a tendency toward a valgus deformity prior to the implantation of the arthroplasty (Figure 5) while the tibial angle approached normality according to the postoperative X-rays (Figure 6).

**Figure 7:**
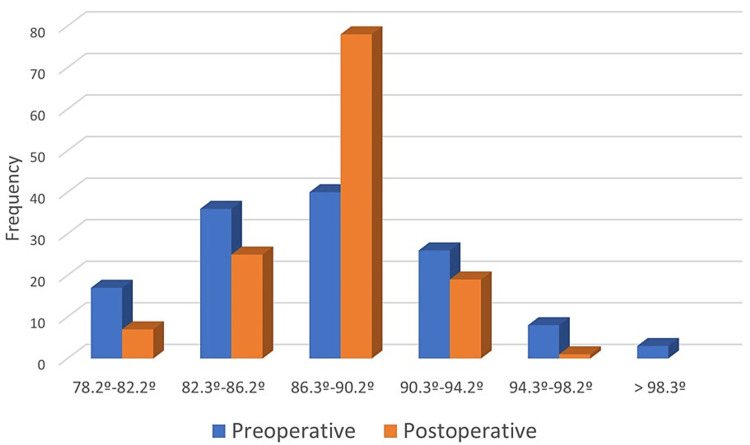
Changes in the tibial axis after arthroplasty The navigation avoided a dispersion of tibial angle values

The proportion of patients with angles within the safe zone, i.e., 90° ± 3°, was compared before and after surgery. The figure rose from 54 (41.54%) patients preoperatively to 79 (60.77%) (p = 0.002) postoperatively, and of the 76 patients whose preoperative angles were not within the safe zone, 46 (60.53%) were seen to be within the “safe zone” after the operation. The patients were randomized into three groups according to the nature of their preoperative tibial deformity: neutral axis (angulation of 90° ± 3°), valgus deformity (angulation smaller than 87°), and varus deformity (angulation larger than 93°). No statistically significant differences were found in any of the groups between the preoperative and the postoperative angulation obtained. After the implantation of the TKA, a trend was observed for tibial angulations to tend toward 90° or a slight valgus deviation, with a reduction of outliers (Figure 7).

It may therefore be considered that navigation avoided a dispersion of tibial angle values and favored the clustering of TCA values.

Statistical analysis

Quantitative variables were described as means, including SD, and proportions as n (%). Student’s t-test for paired data was used to compare pre- and postoperative angulations, and McNemar’s paired proportion test was used to compare rates. The analysis was conducted using the Stata version 16 software (StataCorp LLC, College Station, TX).

## Discussion

The analysis showed that the assistance provided by navigation toward the correct placement of the tibial component in TKA is not significant. Our results indicate that navigation does not offer any advantages over the conventional extramedullary technique, which also prevents the invasion of the intramedullary canal. It must be said, however, that although no differences were observed in terms of TCA, the navigated technique did result in an improvement of the postoperative axis, with more cases within the “safe zone.”

Achieving correct alignment both in the frontal and in the sagittal planes is considered an essential prerequisite for satisfactory clinical and functional results following TKA. The mechanical alignment of the limb should aim at an angle of 180° ± 3° with a “safe zone” at an angle of 90° ± 3° between the mechanical axis and the surface of the proximal tibia. Conversely to the femur, the mechanical axis of the tibia coincides with its anatomical axis, and although the tibial surface in the native knee forms a varus angle of approximately 3°, during bipedal stance and during limb oscillation in unipedal stance, this oblique line becomes parallel to the surface of the upper surface of the talus. In the last few years, several reports have pointed to the high incidence of a constitutional varus among individuals with knee osteoarthritis, with some authors considering that over 30% of males and 17% of females present with a constitutional varus in excess of 3° [5]. This finding has resulted in some surgeons [5] striving to maintain that varus angle following TKA rather than pursuing a neutral mechanical axis at all costs, provided of course that the patients are properly selected. Contrary to kinematic alignment, which allows the tibial axis to range from -6° to +9°, mechanical and functional alignment should pursue a neutral angle of 90° ± 3° [5]. Although this is a controversial issue, a survey among 300 surgeons from 32 countries [6] revealed that most of them preferred neutral tibial alignment yet recognizing that a slight varus was acceptable in the patients with a constitutional varus. For most respondents, the purpose of TKA was to achieve a joint line that was parallel to the ground and a weight-bearing line that ran through the center of the arthroplasty.

Computer-assisted technologies have resulted in an improvement of the axis of the limb and, more importantly, in better functional results following TKA [7,8], without an increase in complications, the rate of readmissions, or the length of hospital stay [9,10]. However, although navigation provides for a more accurate positioning of the femoral component, it has not been shown to improve the rotational position of the tibial component [11-13]. As regards frontal tibial alignment, which is the focus of the present study, no reports exist in the literature indicating whether navigation can result in an improvement. Our series achieved an excellent position, close to 90°, but with no differences with respect to the preoperative frontal tibial axis. Navigation did however bring the tibia closer to the neutral axis and resulted in a higher proportion of cases with a correct postoperative tibial angle and a lower dispersion of cases, without any differences being observed with respect to the degree of previous tibial deformity. Navigation would therefore seem capable of improving frontal tibial alignment and, more importantly, reducing the incidence of outliers, although it cannot be ascertained whether the technique can be considered superior to the use of traditional instrumentation. On the other hand, it cannot be forgotten that navigation is associated with drawbacks such as longer operative times, a steeper learning curve, and higher economic costs.

Table 1 includes some studies that analyze the differences between the intramedullary and the extramedullary techniques [14-20]. According to the different authors, the intramedullary technique results in a correct angulation in 59%-92% of cases, whereas the extramedullary technique gets the angle right in 53%-90% of cases. The former technique leads to a larger proportion of satisfactory results, although only a handful of studies have obtained statistically significant differences [15,16]. Some authors [14] have even found a higher incidence of errors with the intramedullary technique, while metanalyses comparing both techniques [21,22] have found no differences. A survey among surgeons [23] found that three of every four of them preferred the extramedullary technique, yet this preference may be more related to the simplicity of the technique than to a personal decision based on scientific knowledge.

**Table 1:** Differences between intra- and extramedullary alignment of the tibia *This author considers the correct angulation value to be 90° ± 2° **This author considers the correct angulation value to be 90° ± 4° ***Minimally invasive technique IM, intramedullary alignment; EM, extramedullary alignment; CA, correct alignment

Author and year	IM/CA (%)	EM/CA (%)
Dennis et al., 1993 [14]*	60/72	60/88
Maestro et al., 1998 [15]**	61/86.8	55/83.6
Reed et al., 2002 [16]	54/85	46/65
Confalonieri et al., 2005 [17]*	34/85	37/70.8
Chin et al., 2005 [18]	30/91.9	30/90.2
de Kroon et al., 2012 [19]***	23/74	19/63
da Rocha Moreira Rezende et al., 2015 [20]	22/59.1	19/52.6

A few studies have compared manual tibial alignment techniques with navigation. Zahn et al. [4] compared the intramedullary and extramedullary techniques, navigation, and patient-specific instrumentation. Navigation and the intramedullary technique were shown to result in a more accurate reconstruction of the neutral axis of the tibia, with statistically significant differences between the patients’ pre- and postoperative status being observed with both techniques. Moreover, a striking difference was found between the extramedullary technique and navigation regarding the proportion of outliers (13.3% versus 4%). In our study, we set about comparing navigation with the conventional extramedullary technique as both are based on the identification of external anatomical landmarks and both avoid the invasion of the medullary canal.

Table 2 includes a series of studies that compare cases of correct alignment using the extramedullary technique and navigation [4,9,11,17,18,24-26]. Although navigation was shown to be statistically significantly superior in only one study, the rate of correctly aligned cases ranged from 70.2% to 100% with the standard technique as compared with 84%-100% for navigation. Other authors who analyzed the percentage of malaligned cases and the mean alignment deficit [27] found navigation to be associated with slightly better results. The mean TCA obtained in our series following navigation was 87.65°, not too different from the values reported by some authors [28,29] and very far from the 96% reported in others [30]. Similarly, we found no correlation between the value of the TCA and the presence or absence of a previous deformity.

**Table 2:** Tibial component alignment with a manual technique and with navigation *Meta-analysis **Minimally invasive technique MA, manual alignment; NAV, navigation-assisted alignment; CA, correct alignment

Author and year	MA/CA (%)	NAV/CA (%)	P-value
Confalonieri et al., 2005 [17]	37/70.2	38/89.4	
Chin et al., 2005 [18]	25/83.8	28/93.9	0.001
Ensini et al., 2007 [9]	25/100	60/100	
Matziolis et al., 2007 [11]	28/84	32/100	
Mason et al., 2007 [24]*	1086/88.9	1163/96	
Kim et al., 2007 [25]	82/93	97/84	0.188
Dutton et al., 2008 [26]**	56/88	52/92	0.53
Zahn et al., 2020 [4]	75/87.9	75/89.4	

The main limitations of our study include the small sample size, which may have been responsible for the small statistical difference between the pre- and postoperative alignment values of the tibial component. Also, we did not evaluate a group of patients where alignment had been achieved by means of conventional techniques, which would have allowed a comparison of those results with those of patients operated with a navigated technique, because reports on the extramedullary technique are plentiful and well-established in the literature. Although alignment is certainly decisive, it is not the only factor that plays a role in the success of TKA, making it unadvisable to establish a direct correlation between alignment and clinical results.

## Conclusions

The results of this analysis indicate that navigation does not result in an improvement in the mean frontal alignment of the tibial component in TKA, although it does seem to cluster angulation values together with fewer outliers. A review of the literature shows that the manual extramedullary technique favors the proper placement of the tibial tray and that navigation does not offer any advantages over the standard technique.
